# Phenyl *N*-(1,3-thia­zol-2-yl)carbamate

**DOI:** 10.1107/S1600536809020236

**Published:** 2009-06-06

**Authors:** Jian-Guo Tang, Yong-Zhong Wu, Sheng Bi, Guo-Hua Zhang, Cheng Guo

**Affiliations:** a College of Science, Nanjing University of Technology, Xinmofan Road No. 5 Nanjing, Nanjing 210009, People’s Republic of China; b Department of Applied Chemistry, Nanjing College of Chemical Technology, Geguan Road No. 625 Dachang District Nanjing, Nanjing 210048, People’s Republic of China

## Abstract

In the title compound, C_10_H_8_N_2_O_2_S, the planes of the aromatic rings are oriented at a dihedral angle of 66.69 (3)°. In the crystal structure, inter­molecular N—H⋯N and C—H⋯O inter­actions link the mol­ecules into a two-dimensional network, forming *R*_2_^2^(8) ring motifs. π–π contacts between the thia­zole rings [centroid–centroid distance = 3.535 (1) Å] may further stabilize the structure. A weak C—H⋯π inter­action is also found.

## Related literature

For a related structure, see: Araujo *et al.* (2006 ▶). For bond-length data, see: Allen *et al.* (1987 ▶). For ring-motifs, see: Bernstein *et al.* (1995 ▶).
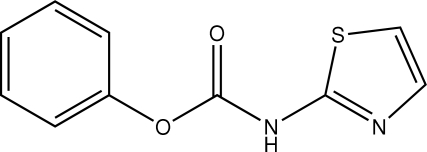


## Experimental

### 

#### Crystal data

C_10_H_8_N_2_O_2_S
*M* *_r_* = 220.24
Monoclinic, 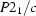

*a* = 5.6430 (11) Å
*b* = 7.3910 (15) Å
*c* = 25.134 (5) Å
β = 91.21 (3)°
*V* = 1048.0 (4) Å^3^
*Z* = 4
Mo *K*α radiation
μ = 0.29 mm^−1^
*T* = 294 K
0.30 × 0.20 × 0.10 mm

#### Data collection

Enraf–Nonius CAD-4 diffractometer
Absorption correction: ψ scan (North *et al.*, 1968 ▶) *T*_min_ = 0.918, *T*_max_ = 0.972
2084 measured reflections
1880 independent reflections
1346 reflections with *I* > 2σ(*I*)
*R*_int_ = 0.027
3 standard reflections frequency: 120 min intensity decay: 1%

#### Refinement

*R*[*F* ^2^ > 2σ(*F* ^2^)] = 0.050
*wR*(*F* ^2^) = 0.160
*S* = 1.00
1880 reflections
136 parameters
H-atom parameters constrained
Δρ_max_ = 0.23 e Å^−3^
Δρ_min_ = −0.28 e Å^−3^

### 

Data collection: *CAD-4 Software* (Enraf–Nonius, 1989 ▶); cell refinement: *CAD-4 Software*; data reduction: *XCAD4* (Harms & Wocadlo, 1995 ▶); program(s) used to solve structure: *SHELXS97* (Sheldrick, 2008 ▶); program(s) used to refine structure: *SHELXL97* (Sheldrick, 2008 ▶); molecular graphics: *ORTEP-3 for Windows* (Farrugia, 1997 ▶) and *PLATON* (Spek, 2009 ▶); software used to prepare material for publication: *SHELXL97* and *PLATON*.

## Supplementary Material

Crystal structure: contains datablocks global, I. DOI: 10.1107/S1600536809020236/hk2701sup1.cif

Structure factors: contains datablocks I. DOI: 10.1107/S1600536809020236/hk2701Isup2.hkl

Additional supplementary materials:  crystallographic information; 3D view; checkCIF report

## Figures and Tables

**Table 1:** Hydrogen-bond geometry (Å, °)

*D*—H⋯*A*	*D*—H	H⋯*A*	*D*⋯*A*	*D*—H⋯*A*
N1—H1*A*⋯N2^i^	0.86	2.01	2.864 (4)	171
C3—H3*A*⋯O2^ii^	0.93	2.46	3.335 (4)	156
C5—H5*A*⋯*Cg*2^iii^	0.93	2.98	3.736 (3)	139

Symmetry codes: (i) 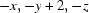; (ii) 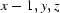; (iii) 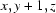. *Cg*2 is the centroid of the S/N2/C8–C10 ring.

**Fig. 1.**
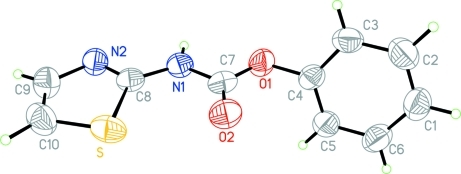
The molecular structure of the title molecule, with the atom-numbering scheme. Displacement ellipsoids are drawn at the 30% probability level.

**Fig. 2.**
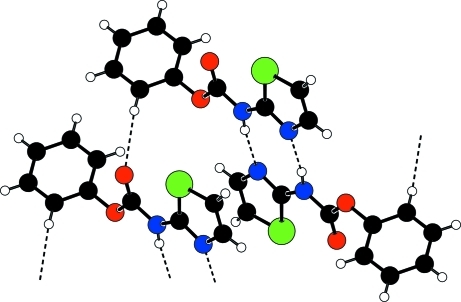
A partial packing diagram of the title compound. Hydrogen bonds are shown as dashed lines.

## supplementary crystallographic information

### Comment

Some derivatives of phenol are important chemical materials. We report herein the crystal structure of the title compound.

In the molecule of the title compound (Fig 1), the bond lengths (Allen *et al.*,  1987) and angles are within normal ranges. Rings A (C1-C6) and B (S/N2/C8-C10) are, of course, planar and they are oriented at a dihedral angle of 66.69 (3)°. Atoms O1, O2, N1, C4, C7, H1A, H9A and H10B are 0.118 (3), -0.063 (3), 0.028 (3), 0.172 (3), 0.023 (3), 0.051 (3), 0.002 (3) and -0.002 (3) Å away from the plane of ring B, respectively.

In the crystal structure, intermolecular N-H···N and C-H···O interactions (Table 1) link the molecules into a two-dimensional network forming *R*_2_^2^(8) ring motifs (Bernstein *et al.*, 1995) (Fig. 2), in which they may be effective in the stabilization of the structure. The π–π contact between the thiazole rings, Cg2—Cg2^i^, [symmetry code: (i) 1 - x, -y, -z, where Cg2 is centroid of the ring B (S/N2/C8-C10)] may further stabilize the structure, with centroid-centroid distance of 3.535 (1) Å. There also exists a weak C—H···π interaction (Table 1).

### Experimental

For the preparation of the title compound, phenyl chloroformate (1.0 ml) was added slowly to a cold solution of thiazol-2-amine (1.0 g) and triethylamine (0.8 ml) in methylene chloride (10 ml) at 273 K. The mixture was then warmed and stirred for 1 h at room temperature. Then, it was washed with water (20 ml), dried and concentrated to give the title compound (yield; 1.3 g) (Araujo *et al.*,  2006). Crystals suitable for X-ray analysis were obtained by slow evaporation of a methanol solution.

### Refinement

H atoms were positioned geometrically, with N-H = 0.86 Å (for NH) and C-H = 0.93 Å for aromatic H and constrained to ride on their parent atoms, with U_iso_(H) = 1.2U_eq_(C,N).

### Crystal data

**Table:**

C_10_H_8_N_2_O_2_S	*F*(000) = 456
*M**_r_* = 220.24	*D*_x_ = 1.396 Mg m^−^^3^
Monoclinic, *P*2_1_/*c*	Mo *K*α radiation, λ = 0.71073 Å
Hall symbol: -P 2ybc	Cell parameters from 25 reflections
*a* = 5.6430 (11) Å	θ = 9–13°
*b* = 7.3910 (15) Å	µ = 0.29 mm^−^^1^
*c* = 25.134 (5) Å	*T* = 294 K
β = 91.21 (3)°	Block, colorless
*V* = 1048.0 (4) Å^3^	0.30 × 0.20 × 0.10 mm
*Z* = 4	

### Data collection

**Table:**

Enraf–Nonius CAD-4 diffractometer	1346 reflections with *I* > 2σ(*I*)
Radiation source: fine-focus sealed tube	*R*_int_ = 0.027
graphite	θ_max_ = 25.3°, θ_min_ = 1.6°
ω/2θ scans	*h* = 0→6
Absorption correction: ψ scan (North *et al.*, 1968)	*k* = 0→8
*T*_min_ = 0.918, *T*_max_ = 0.972	*l* = −30→30
2084 measured reflections	3 standard reflections every 120 min
1880 independent reflections	intensity decay: 1%

### Refinement

**Table:**

Refinement on *F*^2^	Primary atom site location: structure-invariant direct methods
Least-squares matrix: full	Secondary atom site location: difference Fourier map
*R*[*F*^2^ > 2σ(*F*^2^)] = 0.050	Hydrogen site location: inferred from neighbouring sites
*wR*(*F*^2^) = 0.160	H-atom parameters constrained
*S* = 1.00	*w* = 1/[σ^2^(*F*_o_^2^) + (0.07*P*)^2^ + 1.2*P*] where *P* = (*F*_o_^2^ + 2*F*_c_^2^)/3
1880 reflections	(Δ/σ)_max_ < 0.001
136 parameters	Δρ_max_ = 0.23 e Å^−^^3^
0 restraints	Δρ_min_ = −0.28 e Å^−^^3^

### Special details

**Table:**

Geometry. All e.s.d.'s (except the e.s.d. in the dihedral angle between two l.s. planes) are estimated using the full covariance matrix. The cell e.s.d.'s are taken into account individually in the estimation of e.s.d.'s in distances, angles and torsion angles; correlations between e.s.d.'s in cell parameters are only used when they are defined by crystal symmetry. An approximate (isotropic) treatment of cell e.s.d.'s is used for estimating e.s.d.'s involving l.s. planes.
Refinement. Refinement of *F*^2^ against ALL reflections. The weighted *R*-factor *wR* and goodness of fit *S* are based on *F*^2^, conventional *R*-factors *R* are based on *F*, with *F* set to zero for negative *F*^2^. The threshold expression of *F*^2^ > σ(*F*^2^) is used only for calculating *R*-factors(gt) *etc*. and is not relevant to the choice of reflections for refinement. *R*-factors based on *F*^2^ are statistically about twice as large as those based on *F*, and *R*- factors based on ALL data will be even larger.

### Fractional atomic coordinates and isotropic or equivalent isotropic displacement parameters (Å^2^)

**Table:**

	*x*	*y*	*z*	*U*_iso_*/*U*_eq_	
S	0.50888 (16)	0.91653 (14)	0.09893 (4)	0.0578 (3)	
O1	−0.0749 (4)	1.3216 (4)	0.10691 (9)	0.0584 (7)	
O2	0.2490 (4)	1.1925 (4)	0.14685 (10)	0.0604 (7)	
N1	0.0988 (5)	1.0912 (4)	0.06773 (11)	0.0522 (8)	
H1A	−0.0156	1.1050	0.0449	0.063*	
N2	0.2450 (5)	0.8574 (4)	0.01638 (11)	0.0535 (8)	
C1	−0.1494 (7)	1.7169 (6)	0.22167 (16)	0.0653 (11)	
H1B	−0.1690	1.8072	0.2470	0.078*	
C2	−0.3070 (7)	1.5775 (6)	0.21820 (16)	0.0700 (12)	
H2B	−0.4336	1.5728	0.2413	0.084*	
C3	−0.2801 (6)	1.4432 (5)	0.18072 (15)	0.0583 (10)	
H3A	−0.3880	1.3484	0.1781	0.070*	
C4	−0.0921 (6)	1.4522 (5)	0.14755 (13)	0.0476 (8)	
C5	0.0675 (7)	1.5907 (5)	0.15058 (15)	0.0592 (10)	
H5A	0.1945	1.5943	0.1276	0.071*	
C6	0.0395 (8)	1.7244 (6)	0.18762 (16)	0.0666 (11)	
H6A	0.1470	1.8196	0.1899	0.080*	
C7	0.1061 (6)	1.2010 (5)	0.11072 (14)	0.0494 (9)	
C8	0.2638 (6)	0.9587 (5)	0.05834 (13)	0.0446 (8)	
C9	0.4328 (7)	0.7386 (5)	0.01506 (16)	0.0617 (10)	
H9A	0.4494	0.6552	−0.0123	0.074*	
C10	0.5867 (7)	0.7498 (6)	0.05473 (17)	0.0651 (11)	
H10B	0.7206	0.6772	0.0585	0.078*	

### Atomic displacement parameters (Å^2^)

**Table:**

	*U*^11^	*U*^22^	*U*^33^	*U*^12^	*U*^13^	*U*^23^
S	0.0439 (5)	0.0727 (7)	0.0563 (6)	−0.0038 (5)	−0.0113 (4)	0.0105 (5)
O1	0.0530 (14)	0.0639 (17)	0.0574 (15)	0.0055 (13)	−0.0160 (12)	−0.0165 (13)
O2	0.0554 (15)	0.0714 (18)	0.0536 (15)	−0.0004 (13)	−0.0156 (12)	−0.0085 (13)
N1	0.0431 (15)	0.0635 (19)	0.0493 (16)	0.0000 (15)	−0.0130 (13)	−0.0135 (15)
N2	0.0532 (17)	0.0526 (17)	0.0542 (17)	0.0043 (15)	−0.0076 (14)	−0.0044 (15)
C1	0.066 (3)	0.072 (3)	0.058 (2)	0.010 (2)	−0.0082 (19)	−0.014 (2)
C2	0.053 (2)	0.094 (3)	0.063 (2)	0.003 (2)	0.0069 (19)	−0.007 (2)
C3	0.0443 (19)	0.067 (3)	0.064 (2)	−0.0096 (18)	−0.0019 (17)	−0.002 (2)
C4	0.0482 (19)	0.049 (2)	0.0453 (19)	−0.0013 (16)	−0.0090 (15)	−0.0020 (16)
C5	0.057 (2)	0.065 (3)	0.055 (2)	−0.011 (2)	0.0101 (17)	−0.0055 (19)
C6	0.072 (3)	0.062 (2)	0.066 (3)	−0.016 (2)	−0.003 (2)	−0.009 (2)
C7	0.0454 (19)	0.052 (2)	0.051 (2)	−0.0129 (17)	−0.0057 (16)	−0.0003 (17)
C8	0.0433 (18)	0.0499 (19)	0.0403 (18)	−0.0062 (16)	−0.0056 (14)	0.0051 (15)
C9	0.068 (2)	0.056 (2)	0.062 (2)	0.007 (2)	−0.0016 (19)	−0.0001 (19)
C10	0.054 (2)	0.061 (2)	0.080 (3)	0.0108 (19)	0.001 (2)	0.017 (2)

### Geometric parameters (Å, °)

**Table:**

S—C10	1.722 (4)	C1—H1B	0.9300
S—C8	1.729 (3)	C2—C3	1.379 (5)
O1—C4	1.410 (4)	C2—H2B	0.9300
O1—C7	1.358 (4)	C3—C4	1.364 (5)
O2—C7	1.203 (4)	C3—H3A	0.9300
N1—C7	1.351 (4)	C4—C5	1.365 (5)
N1—C8	1.375 (4)	C5—C6	1.369 (5)
N1—H1A	0.8600	C5—H5A	0.9300
N2—C8	1.296 (4)	C6—H6A	0.9300
N2—C9	1.377 (5)	C9—C10	1.311 (6)
C1—C2	1.363 (6)	C9—H9A	0.9300
C1—C6	1.382 (6)	C10—H10B	0.9300
			
C10—S—C8	87.71 (18)	C4—C5—C6	119.6 (4)
C7—O1—C4	117.5 (2)	C4—C5—H5A	120.2
C7—N1—C8	123.7 (3)	C6—C5—H5A	120.2
C7—N1—H1A	118.1	C5—C6—C1	119.6 (4)
C8—N1—H1A	118.1	C5—C6—H6A	120.2
C2—C1—C6	120.1 (4)	C1—C6—H6A	120.2
C2—C1—H1B	120.0	O2—C7—N1	125.5 (3)
C6—C1—H1B	120.0	O2—C7—O1	125.4 (3)
C8—N2—C9	109.8 (3)	N1—C7—O1	109.1 (3)
C1—C2—C3	120.4 (4)	N2—C8—N1	120.5 (3)
C1—C2—H2B	119.8	N2—C8—S	115.2 (3)
C3—C2—H2B	119.8	N1—C8—S	124.3 (2)
C4—C3—C2	118.7 (4)	C10—C9—N2	116.0 (4)
C4—C3—H3A	120.6	C10—C9—H9A	122.0
C2—C3—H3A	120.6	N2—C9—H9A	122.0
C3—C4—C5	121.5 (3)	C9—C10—S	111.2 (3)
C3—C4—O1	118.4 (3)	C9—C10—H10B	124.4
C5—C4—O1	119.9 (3)	S—C10—H10B	124.4
			
C6—C1—C2—C3	−0.2 (6)	C4—O1—C7—O2	2.5 (5)
C1—C2—C3—C4	0.5 (6)	C4—O1—C7—N1	−178.0 (3)
C2—C3—C4—C5	−0.3 (6)	C9—N2—C8—N1	178.6 (3)
C2—C3—C4—O1	−175.6 (3)	C9—N2—C8—S	−0.2 (4)
C7—O1—C4—C3	−112.5 (4)	C7—N1—C8—N2	179.5 (3)
C7—O1—C4—C5	72.1 (4)	C7—N1—C8—S	−1.8 (5)
C3—C4—C5—C6	−0.1 (6)	C10—S—C8—N2	0.1 (3)
O1—C4—C5—C6	175.2 (3)	C10—S—C8—N1	−178.6 (3)
C4—C5—C6—C1	0.3 (6)	C8—N2—C9—C10	0.2 (5)
C2—C1—C6—C5	−0.2 (6)	N2—C9—C10—S	−0.2 (5)
C8—N1—C7—O2	−3.3 (6)	C8—S—C10—C9	0.0 (3)
C8—N1—C7—O1	177.2 (3)		

### Hydrogen-bond geometry (Å, °)

**Table:**

*D*—H···*A*	*D*—H	H···*A*	*D*···*A*	*D*—H···*A*
N1—H1A···N2^i^	0.86	2.01	2.864 (4)	171
C3—H3A···O2^ii^	0.93	2.46	3.335 (4)	156
C5—H5A···Cg2^iii^	0.93	2.98	3.736 (3)	139

Symmetry codes: (i) −*x*, −*y*+2, −*z*; (ii) *x*−1, *y*, *z*; (iii) *x*, *y*+1, *z*.
